# BTLA/HVEM Signaling: Milestones in Research and Role in Chronic Hepatitis B Virus Infection

**DOI:** 10.3389/fimmu.2019.00617

**Published:** 2019-03-29

**Authors:** Xueping Yu, Yijuan Zheng, Richeng Mao, Zhijun Su, Jiming Zhang

**Affiliations:** ^1^Department of Infectious Diseases, First Hospital of Quanzhou, Fujian Medical University, Quanzhou, China; ^2^Department of Infectious Diseases, Huashan Hospital, Fudan University, Shanghai, China

**Keywords:** B and T lymphocyte attenuator, herpes virus entry mediator, hepatitis B virus, milestones, T lymphocyte

## Abstract

B- and T-lymphocyte attenuator (BTLA) is an immune-regulatory receptor, similar to CTLA-4 and PD-1, and is mainly expressed on B-, T-, and all mature lymphocyte cells. Herpes virus entry mediator (HVEM)-BTLA plays a critical role in immune tolerance and immune responses which are areas of intense research. However, the mechanisms of the BTLA and the BTLA/HVEM signaling pathway in human diseases remain unclear. This review describes the research milestones of BTLA and HVEM in chronological order and their role in chronic HBV infection.

## Introduction

Lymphocyte activation is either triggered by the binding of an antigen to its T-cell receptor (TCR) or B-cell receptor (BCR), or by a co-stimulatory or co-inhibitory molecule. T-cells require a co-stimulatory or co-inhibitory molecule for activation, and the quality of T-cell activation is determined by multiple co-signaling molecules. These co-signaling molecules exert both positive stimulatory and negative regulatory functions, and act in a coordinated fashion to maintain homeostasis in the body (1). Co-signaling molecules can be classified into two major families based on their structure. The first is the CD28 immunoglobulin (Ig) superfamily (IgSF), which includes CD28, cytotoxic T-lymphocyte antigen-4 (CTLA-4), inducible costimulatory molecule (ICOS), programmed death-1 (PD-1), and B and T-lymphocyte attenuator (BTLA); and the second is the tumor necrosis factor receptor (TNFR) superfamily (TNFRSF) (2), which includes CD27, CD30, 4-1BB, herpesvirus entry mediator (HVEM), CD40, and OX40 (Table 1). Similar to PD-1 and CTLA-4, BTLA inhibits T-cell reactions and cytokine production. Studies on hepatitis B virus (HBV) infection revealed that BTLA is highly expressed in virus-specific T-cells, which have a potent inhibitory effect on events such as T-cell proliferation and cytokine secretion. In this review, we discuss the biological characteristics of BTLA and its ligand and explore their role in chronic HBV infection.

**Table 1:** Co-stimulatory and inhibitory receptors of the immunoglobulin superfamily and TNFR family.

**Inhibitory molecules**			**Co-stimulatory molecules**		
**Family**	**Molecule**	**Ligand**	**Family**	**Molecule**	**Ligand**
IgSF	PD-1	PDL1	IgSF	CD28	CD80
		PDL2			CD86
	CTLA-4	CD80		ICOS	ICOSL
		CD8	TNFRSF	HVEM	BTLA
	BTLA	HVEM			LIGHT
	Tim3	Galectin-9		4-1BB	4-1BBL
	TIGIT	PVR		OX40	OX40L
		CD155		CD27	CD70
		CD112		CD30	CD30L
	Lag3	MHCII		CD40	CD40L
	CD160	HVEM		GITR	GITRL

## Chronological Milestones in Btla Research

BTLA, also known as CD272, was first discovered by genetic screening in 2003 for its ability to inhibit Th1 cell expression (3). It is the third new member of the CD28 family discovered after PD1 and CTLA-4. In 2005, HVEM was identified as the specific ligand of BTLA (4). HVEM belongs to the TNFR family and not to the Ig family, thus shattering the perspective that receptors exclusively bind with ligands belonging to the same family. In the same year, the herpes simplex virus type 1 glycoprotein D (HSV1 gD) was found to bind to HVEM from the crystal structure of the BTLA-HVEM complex (5). In the subsequent year, the structure, distribution, biological characteristics, and other aspects of BTLA and HVEM were summarized in a review by Murphy et al. (6), who also found a third motif, Grb-2, in the cytoplasmic domain of BTLA that recruits the PI3K-subunit p85, thus, leading to stimulation of the PI3K signaling pathway and subsequent T-cell activation (7). In 2008, the receptors HSV1 gD, LIGHT (also known as tumor necrosis factor superfamily 14) and CD160, were found to constitute the CD160/BTLA/LIGHT/HVEM signaling regulatory network and to share the same ligand HVEM as BTLA (8). The interactions within the CD160/BTLA/LIGHT/HVEM signaling regulatory network were summarized in the 2009 review by Cai and Freeman (9). Specific BTLA antibody clones such as 6F7 and 6H6 targeting the BTLA-HVEM pathway were summarized in the review by Crawford and Wherry (10). Between 2006 and 2010, the roles and mechanisms of BTLA and its ligands in human diseases [organ transplantation (11), intestinal inflammation (12), rheumatoid arthritis (13), and cancer (14)] and animal models [experimental cerebral malaria (15), mouse pancreatic transplantation (16)] were reported. In 2010, Murphy et al. extensively reviewed the biological characteristics and functional mechanisms of BTLA and its ligands and discussed newer findings (17). A new anticancer therapy based on the blockade of the BTLA signaling pathway was proposed next, which signaled the beginning of a new chapter in cancer intervention (18).

**Figure 1:**
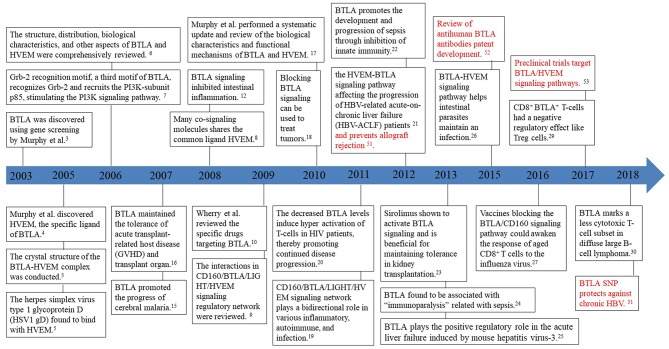
Timeline of milestones in BTLA research.

Since HVEM could interact with many co-signaling molecules, Kronenberg et al. proposed that the CD160/BTLA/LIGHT/HVEM signaling regulatory network plays a bidirectional regulatory role in various inflammatory, autoimmune, and infection immune reactions (19). Decreased BTLA levels could induce hyper-activation of T-lymphocytes in HIV patients thereby promoting disease progression (20). In 2012, the HVEM-BTLA signaling pathway was found to be upregulated in the hepatic tissue of HBV-related acute-on-chronic liver failure (HBV-ACLF) patients, promoting disease progression (21). In addition, BTLA was also found to promote the development and progression of sepsis through inhibition of the innate immune response (22). In 2013, sirolimus was identified to promote the inhibitory effects of BTLA thereby enabling immune tolerance in kidney allograft (23). BTLA was reported to be a crucial molecular marker in “immunoparalysis” associated with sepsis (24), and it was shown to play a positive regulatory role in viral diseases; for example, mouse hepatitis virus-3 (MHV3) could induce BTLA signaling and cause acute liver failure through phagocyte activation and secretion of the inflammatory molecules TNF-α and FGL2 (25). This study advances our understanding of the conditions that determine the negative or positive regulatory functions of BTLA in humans. In 2015, the BTLA-HVEM signaling pathway was reported to help intestinal parasites (especially *Strongyloides stercoralis*) maintain an infection (26). In the following year, vaccines blocking the BTLA/CD160 signaling pathway were shown to activate the response of aged CD8^+^ T cells to the influenza virus (27). Moreover, dendritic cells (DCs) were demonstrated to induce extrathymic T-cell tolerance in peripheral Treg cells through the BTLA-HVEM signaling pathway (28). In 2017, Shen et al. found that the CD8^+^ BTLA^+^ T-cells isolated lymphocytes from the liver tissue of chronic hepatitis B patients had a negative regulatory effect on Treg cells that helped HBV to avoid immune clearance (29). In 2018, BTLA was elucidated as a marker of a less cytotoxic T-cell subset in diffuse large B-cell lymphoma (30). Our summary listing the research milestones on the BTLA signaling pathway in chronological order aims to distill the information from previous findings and provide more explicit research directions for future studies (Figure 1).

## Biological Characteristics Of BTLA

### Structure and Distribution of BTLA

The human BTLA gene, located on chromosome 3 at 3q13, comprises 5 exons with a total length of 870 bp and 3 mRNA splice variants, which encode functional proteins that can be transcribed. Of note, a single nucleotide polymorphism (SNP) of BTLA, rs76844316, was reported to protect against chronic hepatitis B infection (31). BTLA is a type I transmembrane glycoprotein comprising 289 amino acids. Its protein structure is similar to those of CTLA-4 and PD-1 and includes an extracellular domain, transmembrane domain, and cytoplasmic domain (32). The cytoplasmic domain contains three conserved signals: a growth factor receptor-bound protein-2 (Grb-2) recognition motif, an immunoreceptor tyrosine-based inhibitory motif (ITIM), and an immunoreceptor tyrosine-based switch motif (ITSM) (6). ITIM is present in many inhibitory receptors, binding and activating the tyrosine phosphatases SHP-1 and SHP-2, which dephosphorylate tyrosine and inhibit protein tyrosine kinase (PTK)-dependent cell activation (33). The Grb-2 recognition motif recognizes the Grb-2 protein, recruits the PI3K protein subunit p85, and stimulates the PI3K signaling pathway, promoting cell proliferation and survival (7). Thus, the BTLA molecule exerts bidirectional regulatory effects: immunosuppressive effects like those on CTLA-4 and PD-1 proteins, and positive stimulatory effects like those on CD28 and ICOS proteins.

BTLA is widely expressed in the spleen, thymus, and lymph nodes and has relatively low expression or is even undetectable in the liver, kidney, heart, brain, and other organs. BTLA is constitutively expressed in the CD4/CD8 single-positive T-cells in the mouse thymus (34). Additionally, BTLA is highly expressed in the B-lymphocytes, splenic macrophages, and bone marrow-derived dendritic cells (35, 36).

### BTLA Ligands

HVEM, a BTLA-specific receptor, was discovered in 2005 (4). HVEM is expressed in peripheral T- and B-cells, highly expressed in resting T cells, immature B cells, and memory B cells, but downregulated in activated T and B cells. Additionally, it is widely expressed in monocytes, dendritic cells, Treg cells, neutrophils, and NK cells (37, 38). HVEM binds with many co-signaling molecules, both co-stimulatory and co-inhibitory. The roles that both types of signaling molecules play in signaling pathways also differ and are known as the “molecular switch” models of activation and inhibition. Binding of HVEM to LIGHT or LIGHT-α exerts a positive stimulatory effect, stimulating lymphocyte proliferation, activation, and inducing inflammatory reactions; thus, providing a second stimulatory signal for T cell activation (4, 39). Binding of HVEM to BTLA and CD160 exerts an adverse regulatory effect, inhibiting T- and B-lymphocyte activation and proliferation and binding of HVEM to HSV-gD, which can promote HSV infection in target cells (4). Taken together, HVEM provides either an inhibitory or activating signal and bi-directionally regulates host immune function.

## Function of BTLA in Immune Cells

### Function of BTLA in T-lymphocytes

Resting T-cells express high levels of BTLA and HVEM, and T cell activation increases or decreases BTLA and HVEM expression, respectively. The inhibition of T-cells by BTLA is stronger than the positive stimulatory effect of HVEM on T-cells and prevents the excessive activation of T-cells (40). Importantly, HVEM and BTLA in naive T-cells form a cis-heterodimeric complex, blocking the external CD160 and other co-signaling molecules from binding to HVEM and stimulating the NF-κB signaling pathway, thereby maintaining T-cell tolerance (41). Other studies have demonstrated that BTLA gene knockdown mice (*Btla*^−/−^) resist immune tolerance induced by high doses of oral or intravenous ovalbumin (OVA) and show increased infiltration by the inflammatory cells in multiple organs, which induces an autoimmune hepatitis-like disease (42, 43). Additionally, Liu et al. could not induce immune tolerance in *Btla*^−/−^ mice injected with large doses of OVA, indicating that BTLA plays a vital role in inducing and maintaining T cell immune tolerance (44).

In addition to inhibiting antigen-specific TCR signaling-mediated T-cell proliferation, activation (CD25, CD38), and cytokine (IL-2, IL-4, and IL-10) production (34), the BTLA molecule also crosslinks HVEM on Treg cells to facilitate their immunosuppressant effects (40). Further, BTLA inhibits IgG production by inhibiting secretion of IL-21 by follicular helper T cells (Tfh) and plays an essential role in the immunomodulation in body fluids (45). γδT cells play an important role in pathogen clearance and the anticancer process. Interestingly, BTLA inhibits γδT cell proliferation, and secretion of IL-17, TNF-α, and other cytokines leading to decreased pathogen clearance and anticancer activity (46, 47).

In some instances, binding of BTLA and HVEM mediates immunosuppressive activity and transduces positive signals that promote the survival of effector T cells (33). Tarun et al. used the vaccinia virus to infect mice and found that the BTLA-HVEM co-signaling system significantly promotes the survival of antiviral effector CD8^+^ T-cells and production of memory cells (48). Competitive stimulation with BTLA antibodies (3C10) can induce IL-10-dependent Treg cell production and helps prolong allogeneic heart transplantation in mice (49). Additionally, BTLA can increase the number and activity of γδT cells and reduce the symptoms of skin inflammation; *Btla*^−/−^ mice have a reduced number of γδT cells and are susceptible to dermatitis. Moreover, BTLA/HVEM crosslinking was observed to suppress T-cell activation thereby preventing allograft rejection (21, 50). Variations of the therapeutic strategy that targets the BTLA-HVEM immune checkpoint pathway using specific antagonist antihuman antibodies has been published in numerous patents, and drugs capable of targeting BTLA-associated signaling pathways such as the HVEM-BTLA-CD160 pathway are currently in preclinical trials (10, 18, 51, 52).

### Function of BTLA in B-lymphocytes

BTLA research has been focused on T-cells, and there are few studies on its function in B-cells. Previous studies have revealed that BTLA is an inhibitory receptor in the BCR signaling pathway. BTLA attenuates the BCR signaling strength by recruiting and phosphorylating the protein tyrosine kinase Syk and downregulating B-cell linker protein, phospholipase E2, and NF-κB (53). Ware et al. suggested that HVEM-BTLA signaling can inhibit CPG-mediated B-cell proliferation and cytokine secretion, and increase stimulatory molecules on their surface; however, this does not affect IL-8 and MIP-1β secretion, indicating that BTLA can partially, but not completely, inhibit B cell function (54). However, studies have also shown that BTLA expression in B cells is decreased in elderly patients, leading to reduced responsiveness to the trivalent influenza vaccine, and an inability to produce useful IgG antibodies and mount effective vaccination responses (55). Thus, BTLA can play bidirectional regulatory roles in specific cases.

### Function of BTLA in Dendritic Cells

Latest research demonstrates that HVEM-BTLA signaling plays an important role in maintaining the stability of the internal environment for DCs. Lymphotoxin beta receptor (LT-βR) signaling can induce DC proliferation, whereas HVEM-BTLA signaling inhibits their proliferation, indicating that HVEM-BTLA signaling can regulate LT-βR signaling by feedback and maintain the stability of the internal environment for DCs (56). Interestingly, an adenoviral infection can cause immature DCs to express high levels of CCR7 and exhibit relatively strong migration ability. However, their immune tolerance is relatively poor, and the overexpression of BTLA promotes the maintenance of immune tolerance in these DCs (57). Additionally, BTLA^+^ DCs in the thymus increase the expression of CD5 in peripheral T-cells through the BTLA-HVEM signaling pathway and promote the differentiation of these CD5^+^T-cells into Treg cells; thus, producing extrathymic T-cell tolerance (28).

### Function of BTLA in Natural Killer T-cells

Like B- and T-lymphocytes, BTLA is expressed in the natural killer T (NKT) cells. Nakajima et al. established that BTLA^−/−^ NKT mice secrete more cytokines (IFN-γ and IL-4) after α-galactosylceramide stimulation and Con A injection compared to wild-type mice, and develop Con A-induced hepatitis more easily(58). However, these phenomena were not observed in BTLA^−/−^NKT^−/−^ mice. When BTLA^−/−^NKT and NKT cells were purified *in vitro* and injected into the NKT^−/−^ mice, mice receiving BTLA^−/−^NKT cells were more susceptible to Con A-induced hepatitis, indicating that BTLA inhibits hepatitis induced by NKT cells. Similarly, Fu et al. also found in a Con A-induced acute hepatitis model that NKT cells inhibit the release of cytokines (IFN-γ, IL-2, and IL-4) and liver tissue damage through upregulation of the HVEM-BTLA signaling pathway (59). Additionally, in mouse models of breast cancer, type I NKT cells express high levels of BTLA, and blocking the BTLA signaling pathway may promote infiltration of tumors by NKT cells and inhibit tumor growth (60).

## Function of BTLA in Chronic HBV Infection

HBV infection severely endangers the health of humans. Globally, there are 240 million patients with HBV infection and every year 0.65 million patients die of HBV-associated end-stage liver diseases, whose leading mortality causes include liver cirrhosis (LC), liver failure (LF), primary hepatic cell carcinoma (HCC). China is an endemic zone for HBV infection, and currently, there are 93 million individuals with chronic HBV infection, with ~20 million chronic hepatitis B (CHB) patients. Thus, HBV infections put a heavy economic burden on the country and its citizens. However, the pathogenic mechanism of chronic HBV infection is not completely understood. Research has demonstrated that the HBVM-BTLA signaling pathway plays an important role in cancer (14, 61), intestinal inflammation (12), autoimmune diseases (4, 13), viral infection (62), transplant rejection (11, 63), and in continuous chronic HBV infection. In this section, we have provided a current summary of the literature review of BTLA's functions in chronic HBV infection.

### Function of BTLA in CHB

The response of HBV-specific T cells (CTL) in CHB patients is extremely weak and can be undetectable. Additionally, the inability to clear HBV leads to continuous infection. Multiple reports suggest that this could be related to an increased expression of T-cell co-inhibitory molecules (e.g., PD-1). However, recent studies demonstrated no significant difference in the peripheral blood expression of BTLA in CD4^+^ and CD8^+^T-cells in CHB patients and healthy individuals, (64, 65) and the expression levels were similar in the 4 subtypes of CD4^+^T and CD8^+^T cells (TEM-RA, Tnaïve, Tcm, and Tem) (64). Although these results suggest that BTLA does not contribute to chronic HBV infection (or CHB immune tolerance); however, there is a difference in BTLA expression levels in the CTL subtypes in the peripheral blood and liver tissue of CHB patients. In the peripheral blood, BTLA is primarily expressed in the Tcm subtype of T-lymphocytes, whereas BTLA in the liver tissue is primarily expressed in the Tem subtype. This difference may be due to an upregulation of BTLA expression during homing of the peripheral CD8^+^T-cells to the liver that prevents the excessive transition of CD8^+^T cells from the CM stage to the EM stage helping HBV evade immune clearance. Thus, it is believed that CD8^+^BTLA^+^T cells can negatively regulate Treg cells (29). Critically, during the four different phases of HBV infection [immunotolerant phase, immune clearance phase, non-reactive or minimally (non-) replicative phase, and reactivation phase], the immune reactions produced by HBV are different. Additionally, Zhou et al. established that the frequency of rs76844316 in the G allele of the BTLA gene was decreased in patients with severe CHB, which leads to increased sensitivity to HBV and association with severe disease (31). Therefore, the total BTLA expression level in CHB patients should not be viewed in isolation and BTLA expression in patients at different phases of HBV, across multiple severities of CHB should be analyzed. However, to our knowledge, no such data have been reported.

### Function of BTLA in HBV-LC and HCC

**Figure 2:**
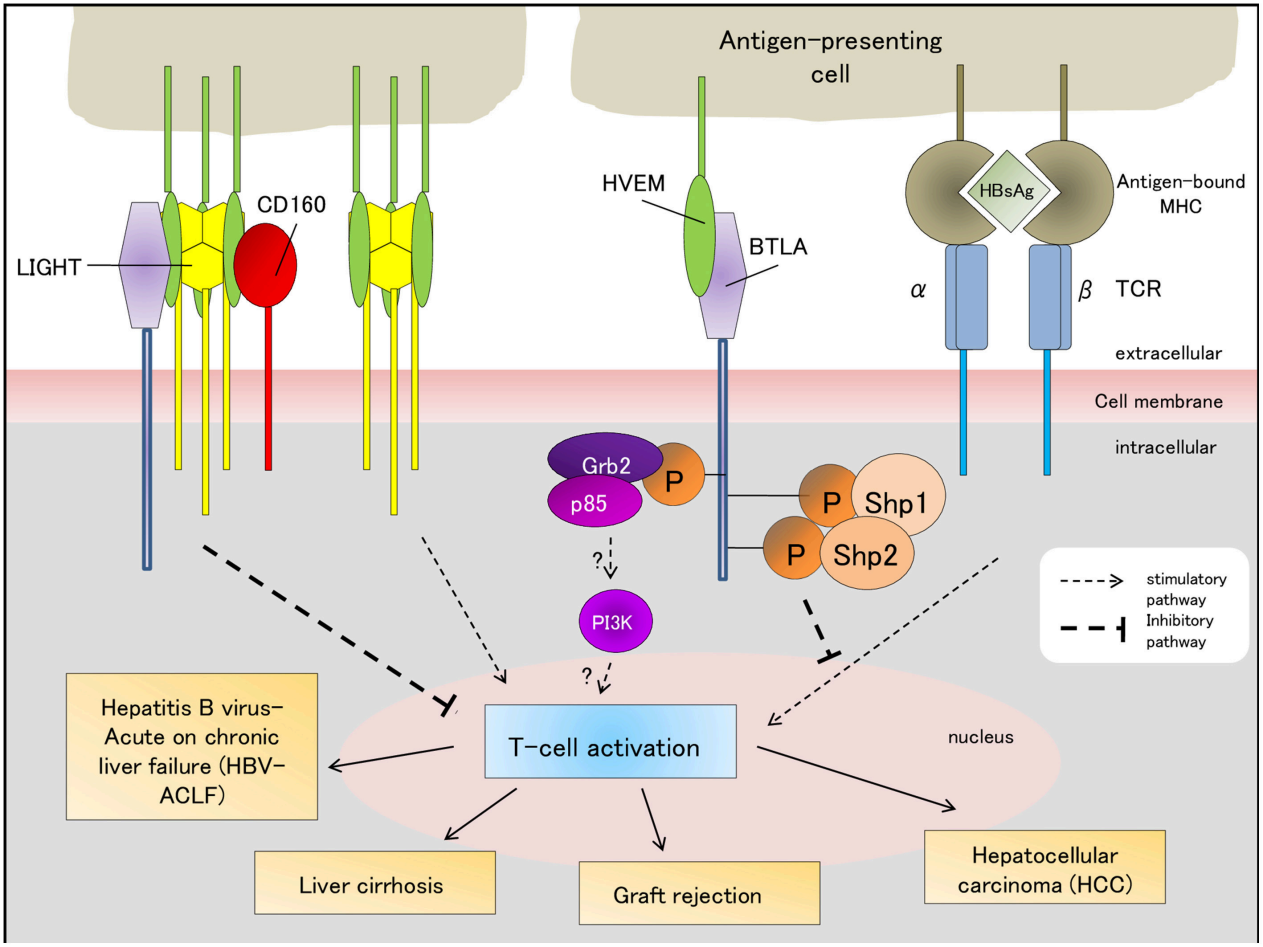
BTLA-associated signaling pathways (stimulatory/inhibitory) regulate the outcomes of HBV-ACLF, liver graft tolerance/rejection, liver cirrhosis and hepatocellular carcinoma. The “?” indicates that the role of the Grb-2 pathway in T-cell-related immune diseases requires further validation and has not been fully characterized yet. HBsAg, Hepatitis B antigen; TCR, T-cell receptor; MCH, major histocompatibility complex.

CHB is a progressive disease, and the Chinese “Guidelines for the Prevention of Chronic Hepatitis B (2015 edition)” indicates that every year, ~2–10% of the CHB patients develop LC and 3–6% of the LC patients further progress into HCC (66). Liao et al. suggested that BTLA expression levels are significantly upregulated during the progression of CHB from HBV-LC to HCC, but the expression levels of other co-signaling molecules (CD28, ICOS, LIGHT) do not change significantly indicating that BTLA plays an important role in the progression of CHB (67). BTLA is expressed at high levels in the peripheral blood of patients with HBV-associated HCC and directly correlates with CD4^+^CD25^+^Treg cells. These findings indicate that BTLA may have a synergistic effect with CD4^+^CD25^+^Treg cells, inhibit T-cell activity and proliferation, and promote the immune evasion of tumors (68). Thus, blocking the BTLA signaling pathway inhibits T-cell function, “awakens” cancer recognition by the immune system, and clears tumor cells. Blockade of the HVEM-BTLA signaling pathway has been developed as a new anticancer method (18, 52) and has led to more anticancer drugs that target BTLA (Figure 2).

### Function of BTLA in HBV-ACLF

Liver failure is categorized as acute liver failure (ALF), subacute liver failure (SALF), acute-on-chronic liver failure (ACLF), and chronic liver failure (CLF) (69). Clinically, the most common form of HBV-associated liver failure is HBV-associated acute-on-chronic liver failure (ACLF) in China, and it has a high mortality rate of 60–80% (70). ACLF was first coined in 1995 describing a condition arising from two simultaneous insults to the liver, one ongoing and one acute (71). The consensus statements of its definition, diagnosis and management were approved at the 2008 Annual Conference of the APASL in Korea (72), which was defined as an increasingly recognized syndrome characterized by an acute deterioration of liver function and organ/system failure (liver, kidney, brain, coagulation, circulation, and/or respiration) (70).

Since ACLF is a complex and dynamic disease, its diagnostic criteria consists of several components: the cause and timeframe of liver disease development and deterioration following an acute insult, whether patients have pre-existing chronic liver disease, the symptoms of liver failure to determine ACLF severity, and how to assess short- (28-day) and long-term (90-day) prognoses (73). HBV infection is one of the causes of acute liver injury, and BTLA/HVEM signaling contributed to HBV-ACLF pathogenesis (21). The timeframe of acute liver disease development after an acute insult has not been rigorously defined, but several studies have reported it to range from 2 to 8 weeks (73). The role of BTLA/HVEM signaling in determining this timeframe has not been characterized yet. BTLA/HVEM signaling pathway has been shown to prevent T-cell activation thereby promoting malignancy (Figure 2) (68). However, blocking this co-inhibitory pathway limited antitumor response against pre-existing tumor cells (74). The association of BTLA/HVEM signaling with the symptoms of liver failure in ACLF has not been elucidated. Although BTLA/HVEM signaling was shown to be implicated in poor HBV-ACLF outcome (21), further research is necessary to determine its potential as a biomarker for both short- and long-term clinical prognosis.

Currently, there are no effective therapeutic measures, and it is critical to search for early diagnostic markers or targets for pharmacological intervention. Upregulation of PD-1/PD-L1 (75) and BTLA/HVEM (21) pathways in the liver tissue of HBV-ACLF patients were reported. Additionally, BTLA is primarily expressed in fibrinogen-like protein-2 and CD68^+^ phagocytes and is not expressed in the liver tissue of CHB patients or healthy individuals. Thus, BTLA has the potential to be a diagnostic marker for HBV-ACLF and provides a theoretical basis for HBV-ACLF immunotherapy.

## Concluding Remarks

Since the discovery of BTLA in 2003, multiple studies have established that the HVEM-BTLA signaling pathway plays an essential immunomodulatory role in autoimmune disease, cancer, transplantation, infection, and other diseases. Recent studies on the HVEM-BTLA signaling pathways have unveiled the function and mechanism of BTLA, and targeted anticancer drugs for HVEM-BTLA are emerging. However, whether BTLA participates in inducing functional exhaustion of T-cells and its pathophysiological roles in associated diseases (e.g., HBV-ACLF) remain unknown. It is predicted that studies targeting BTLA will lead to a new revolution in unraveling the immune mechanisms, diagnosis, and treatment of chronic HBV infection.

## Author Contributions

XY and YZ searched, identified and reviewed the literature, and wrote the manuscript. RM made a table, gave critical comments, and revised the manuscript. JZ and ZS identified and reviewed the literature, wrote the manuscript, and revised the manuscript. All authors have made an intellectual contribution to the manuscript and approved the submission.

### Conflict of Interest Statement

The authors declare that the research was conducted in the absence of any commercial or financial relationships that could be construed as a potential conflict of interest.

## References

[1] McGrath MM Najafian N. The role of coinhibitory signaling pathways in transplantation and tolerance. Front Immunol. (2012) 3:47. 10.3389/fimmu.2012.00047 22566929 PMC3342378

[2] del Rio ML Kaye J Rodriguez-Barbosa JI Detection of protein on BTLA low cells and *in vivo* antibody-mediated down-modulation of BTLA on lymphoid and myeloid cells of C57BL/6 and BALB/c BTLA allelic variants. Immunobiology. (2010) 215:570–8. 10.1016/j.imbio.2009.09.008 19837478

[3] Gavrieli M Watanabe N Loftin SK Murphy TL Murphy KM. Characterization of phosphotyrosine binding motifs in the cytoplasmic domain of B and T lymphocyte attenuator required for association with protein tyrosine phosphatases SHP-1 and SHP-2. Biochem Biophys Res Commun. (2003) 312:1236–43. 10.1016/j.bbrc.2003.11.070 14652006

[4] Sedy JR Gavrieli M Potter KG Hurchla MA Lindsley RC Hildner K. B and T lymphocyte attenuator regulates T cell activation through interaction with herpesvirus entry mediator. Nat Immunol. (2005) 6:90–8. 10.1038/ni1144 15568026

[5] Compaan DM Gonzalez LC Tom I Loyet KM Eaton D Hymowitz SG. Attenuating lymphocyte activity: the crystal structure of the BTLA-HVEM complex. J Biol Chem. (2005) 280:39553–61. 10.1074/jbc.M507629200 16169851

[6] Murphy KM Nelson CA Sedy JR. Balancing co-stimulation and inhibition with BTLA and HVEM. Nat Rev Immunol. (2006) 6:671–81. 10.1038/nri1917 16932752

[7] Gavrieli M Murphy KM. Association of Grb-2 and PI3K p85 with phosphotyrosile peptides derived from BTLA. Biochem Biophys Res Commu. (2006) 345:1440–5. 10.1016/j.bbrc.2006.05.036 16725108

[8] Kaye J. CD160 and BTLA: LIGHTs out for CD4+ T cells. Nat Immunol. (2008) 9:122–4. 10.1038/ni0208-122 18204424

[9] Cai G Freeman GJ. The CD160, BTLA, LIGHT/HVEM pathway: a bidirectional switch regulating T-cell activation. Immunol Rev. (2009) 229:244–58. 10.1111/j.1600-065X.2009.00783.x 19426226

[10] Crawford A Wherry EJ. Editorial: therapeutic potential of targeting BTLA. J Leukoc Biol. (2009) 86:5–8. 10.1189/jlb.0209076 19567411

[11] Hurchla MA Sedy JR Murphy KM. Unexpected role of B and T lymphocyte attenuator in sustaining cell survival during chronic allostimulation. J Immunol. (2007) 178:6073–82. 10.4049/jimmunol.178.10.6073 17475832

[12] Steinberg MW Turovskaya O Shaikh RB Kim G McCole DF Pfeffer K. A crucial role for HVEM and BTLA in preventing intestinal inflammation. J Exp Med. (2008) 205:1463–76. 10.1084/jem.20071160 18519647 PMC2413041

[13] Lin SC Kuo CC Chan CH. Association of a BTLA gene polymorphism with the risk of rheumatoid arthritis. J Biomed Sci. (2006) 13:853–60. 10.1007/s11373-006-9113-7 17024343

[14] Wegiel B Bjartell A Culig Z Persson JL. Interleukin-6 activates PI3K/Akt pathway and regulates cyclin A1 to promote prostate cancer cell survival. Int J Cancer. (2008) 122:1521–9. 10.1002/ijc.23261 18027847

[15] Lepenies B Pfeffer K Hurchla MA Murphy TL Murphy KM Oetzel J. Ligation of B and T lymphocyte attenuator prevents the genesis of experimental cerebral malaria. J Immunol. (2007) 179:4093–100. 10.4049/jimmunol.179.6.4093 17785848

[16] Truong W Plester JC Hancock WW Merani S Murphy TL Murphy KM. Combined coinhibitory and costimulatory modulation with anti-BTLA and CTLA4Ig facilitates tolerance in murine islet allografts. Am J Transplant. (2007) 7:2663–74. 10.1111/j.1600-6143.2007.01996.x 17983390

[17] Murphy TL Murphy KM. Slow down and survive: enigmatic immunoregulation by BTLA and HVEM. Annu Rev Immunol. (2010) 28:389–411. 10.1146/annurev-immunol-030409-101202 20307212

[18] Paulos CM June CH. Putting the brakes on BTLA in T cell-mediated cancer immunotherapy. J Clin Invest. (2010) 120:76–80. 10.1172/JCI41811 20038807 PMC2798708

[19] Shui JW Steinberg MW Kronenberg M. Regulation of inflammation, autoimmunity, and infection immunity by HVEM-BTLA signaling. J Leukoc Biol. (2011) 89:517–23. 10.1189/jlb.0910528 21106644 PMC3058819

[20] Zhang Z Xu X Lu J Zhang S Gu L Fu J. B and T lymphocyte attenuator down-regulation by HIV-1 depends on type I interferon and contributes to T-cell hyperactivation. J Infect Dis. (2011) 203:1668–78. 10.1093/infdis/jir165 21592997 PMC3988446

[21] Xu H Cao D Guo G Ruan Z Wu Y Chen Y. The intrahepatic expression and distribution of BTLA and its ligand HVEM in patients with HBV-related acute-on-chronic liver failure. Diagn Pathol. (2012) 7:142. 10.1186/1746-1596-7-142 23067542 PMC3488509

[22] Shubin NJ Chung CS Heffernan DS Irwin LR Monaghan SF Ayala A. BTLA expression contributes to septic morbidity and mortality by inducing innate inflammatory cell dysfunction. J Leukoc Biol. (2012) 92:593–603. 10.1189/jlb.1211641 22459947 PMC3427605

[23] Bai Y Shi Y Li Y Cai B Zou Y Wang L. Sirolimus-based regimen promotes inhibitory costimulatory signal of HVEM/BTLA/CD160/LIGHT pathway in allo-renal recipients. Trans Immunol. (2013) 28:38–47. 10.1016/j.trim.2012.11.005 23165214

[24] Sherwood ER Hotchkiss RS. BTLA as a biomarker and mediator of sepsis-induced immunosuppression. Crit Care. (2013) 17:1022. 10.1186/cc13143 24321139 PMC4059397

[25] Yang C Chen Y Guo G Li H Cao D Xu H. Expression of B and T lymphocyte attenuator (BTLA) in macrophages contributes to the fulminant hepatitis caused by murine hepatitis virus strain-3. Gut. (2013) 62:1204–13. 10.1136/gutjnl-2012-302239 22637698

[26] Breloer M Hartmann W Blankenhaus B Eschbach ML Pfeffer K Jacobs T. Cutting edge: the BTLA-HVEM regulatory pathway interferes with protective immunity to intestinal helminth infection. J Immunol. (2015) 194:1413–6. 10.4049/jimmunol.1402510 25595777

[27] Dawany N Parzych EM Showe LC Ertl HC. Age-related changes in the gene expression profile of antigen-specific mouse CD8+ T cells can be partially reversed by blockade of the BTLA/CD160 pathways during vaccination. Aging (Albany NY). (2016) 8:3272–97. 10.18632/aging.101105 27922818 PMC5270668

[28] Jones A Bourque J Kuehm L Opejin A Teague Ryan M Gross C. Immunomodulatory functions of BTLA and HVEM govern induction of extrathymic regulatory T cells and tolerance by dendritic cells. Immunity. (2016) 45:1066–77. 10.1016/j.immuni.2016.10.008 27793593 PMC5112132

[29] Wang H Wu B Li L Hu L Lin J Jiang C. Hepatic expansion of virus-specific CD8(+)BTLA(+) T cells with regulatory properties in chronic hepatitis B virus infection. Cell Immunol. (2017) 311:36–45. 10.1016/j.cellimm.2016.10.002 27743606

[30] Quan L Lan X Meng Y Guo X Guo Y Zhao L. BTLA marks a less cytotoxic T-cell subset in diffuse large B-cell lymphoma with high expression of checkpoints. Exp Hematol. (2018) 60:47–56.e1. 10.1016/j.exphem.2018.01.003 29353075

[31] Tang J Fei J Gu C Liu W Li M Zhou C. The influence of B and T lymphocyte attenuator genetic variants on susceptibility to chronic hepatitis B virus infection. Cell Physiol Biochem. (2018) 45:2540–7. 10.1159/000488272 29558758

[32] Watanabe N Gavrieli M Sedy JR Yang J Fallarino F Loftin SK. BTLA is a lymphocyte inhibitory receptor with similarities to CTLA-4 and PD-1. Nat Immunol. (2003) 4:670–9. 10.1038/ni944 12796776

[33] Sakoda Y Park JJ Zhao Y Kuramasu A Geng D Liu Y. Dichotomous regulation of GVHD through bidirectional functions of the BTLA-HVEM pathway. Blood. (2011) 117:2506–14. 10.1182/blood-2010-08-301325 21220749 PMC3062413

[34] Han P Goularte OD Rufner K Wilkinson B Kaye J. An inhibitory Ig superfamily protein expressed by lymphocytes and APCs is also an early marker of thymocyte positive selection. J Immunol. (2004) 172:5931–9. 10.4049/jimmunol.172.10.5931 15128774

[35] Hurchla MA Sedy JR Gavrieli M Drake CG Murphy TL Murphy KM. B and T lymphocyte attenuator exhibits structural and expression polymorphisms and is highly Induced in anergic CD4+ T cells. J Immunol. (2005) 174:3377–85. 10.4049/jimmunol.174.6.3377 15749870

[36] Pilat N Sayegh MH Wekerle T. Costimulatory pathways in transplantation. Sem Immunol. (2011) 23:293–303. 10.1016/j.smim.2011.04.002 21616680 PMC3203219

[37] Nurieva RI Chung Y Hwang D Yang XO Kang HS Ma L Generation of T follicular helper cells is mediated by interleukin-21 but independent of T helper 1, 2, or 17 cell lineages. Immunity. (2008) 29:138–49. 10.1016/j.immuni.2008.05.009 18599325 PMC2556461

[38] Steinberg MW Cheung TC Ware CF. The signaling networks of the herpesvirus entry mediator (TNFRSF14) in immune regulation. Immunol Rev. (2011) 244:169–87. 10.1111/j.1600-065X.2011.01064.x 22017438 PMC3381650

[39] del Rio ML Lucas CL Buhler L Rayat G Rodriguez-Barbosa JI. HVEM/LIGHT/BTLA/CD160 cosignaling pathways as targets for immune regulation. J Leukoc Biol. (2010) 87:223–35. 10.1189/jlb.0809590 20007250

[40] Tao R Wang L Murphy KM Fraser CC Hancock WW. Regulatory T Cell Expression of herpesvirus entry mediator suppresses the function of B and T lymphocyte attenuator-positive effector T cells. J Immunol. (2008) 180:6649–55. 10.4049/jimmunol.180.10.6649 18453584

[41] Cheung TC Oborne LM Steinberg MW Macauley MG Fukuyama S Sanjo H. T cell intrinsic heterodimeric complexes between HVEM and BTLA determine receptivity to the surrounding microenvironment. J Immunol. (2009) 183:7286–96. 10.4049/jimmunol.0902490 19915044 PMC2865256

[42] Oya Y Watanabe N Kobayashi Y Owada T Oki M Ikeda K. Lack of B and T lymphocyte attenuator exacerbates autoimmune disorders and induces Fas-independent liver injury in MRL-lpr/lpr mice. Int Immunol. (2011) 23:335–44. 10.1093/intimm/dxr017 21521881

[43] Oya Y Watanabe N Owada T Oki M Hirose K Suto A. Development of autoimmune hepatitis-like disease and production of autoantibodies to nuclear antigens in mice lacking B and T lymphocyte attenuator. Arthritis Rheum. (2008) 58:2498–510. 10.1002/art.23674 18668554 PMC2782777

[44] Liu X Alexiou M Martin-Orozco N Chung Y Nurieva RI Ma L. Cutting edge: a critical role of B and T lymphocyte attenuator in peripheral T cell tolerance induction. J Immunol. (2009) 182:4516–20. 10.4049/jimmunol.0803161 19342624 PMC2767106

[45] Kashiwakuma D Suto A Hiramatsu Y Ikeda K Takatori H Suzuki K. B and T lymphocyte attenuator suppresses IL-21 production from follicular Th cells and subsequent humoral immune responses. J Immunol. (2010) 185:2730–6. 10.4049/jimmunol.0903839 20660710

[46] Bekiaris V Sedy JR Macauley MG Rhode-Kurnow A Ware CF. The inhibitory receptor BTLA controls gammadelta T cell homeostasis and inflammatory responses. Immunity. (2013) 39:1082–94. 10.1016/j.immuni.2013.10.017 24315996 PMC3909738

[47] Gertner-Dardenne J Fauriat C Olive D. BTLA, a key regulator of Vgamma9Vdelta2 T-cell proliferation. Oncoimmunology. (2013) 2:e25853. 10.4161/onci.25853 24244908 PMC3825728

[48] Flynn R Hutchinson T Murphy KM Ware CF Croft M Salek-Ardakani S. CD8 T cell memory to a viral pathogen requires trans cosignaling between HVEM and BTLA. PLoS ONE. (2013) 8:e77991. 10.1371/journal.pone.0077991 24205056 PMC3812147

[49] Uchiyama M Jin X Matsuda H Bashuda H Imazuru T Shimokawa T. An agonistic anti-BTLA mAb (3C10) induced generation of IL-10-dependent regulatory CD4+ T cells and prolongation of murine cardiac allograft. Transplantation. (2014) 97:301–9. 10.1097/01.TP.0000438204.96723.8b 24448587

[50] Hobo W Norde WJ Schaap N Fredrix H Maas F Schellens K. B and T lymphocyte attenuator mediates inhibition of tumor-reactive CD8+ T cells in patients after allogeneic stem cell transplantation. J Immunol. (2012) 189:39–49. 10.4049/jimmunol.1102807 22634623

[51] Collin M. Immune checkpoint inhibitors: a patent review (2010–2015). Expert Opin Ther Pat. (2016) 26:555–64. 10.1080/13543776.2016.1176150 27054314

[52] Torphy RJ Schulick RD Zhu Y. Newly emerging immune checkpoints: promises for future cancer therapy. Int J Mol Sci. (2017) 18:E2642. 10.3390/ijms18122642 29211042 PMC5751245

[53] Vendel AC Calemine-Fenaux J Izrael-Tomasevic A Chauhan V Arnott D Eaton DL. B and T lymphocyte attenuator regulates B cell receptor signaling by targeting Syk and BLNK. J Immunol. (2009) 182:1509–17. 10.4049/jimmunol.182.3.1509 19155498

[54] Thibult ML Rivals JP Mamessier E Gertner-Dardenne J Pastor S Speiser DE. CpG-ODN-induced sustained expression of BTLA mediating selective inhibition of human B cells. J Mol Med. (2013) 91:195–205. 10.1007/s00109-012-0943-7 22903545

[55] Kannan S Kurupati RK Doyle SA Freeman GJ Schmader KE Ertl HC. BTLA expression declines on B cells of the aged and is associated with low responsiveness to the trivalent influenza vaccine. Oncotarget. (2015) 6:19445–55. 10.18632/oncotarget.4597 26277622 PMC4637297

[56] De Trez C Schneider K Potter K Droin N Fulton J Norris PS. The inhibitory HVEM-BTLA pathway counter regulates lymphotoxin receptor signaling to achieve homeostasis of dendritic cells. J Immunol. (2008) 180:238–48. 10.4049/jimmunol.180.1.238 18097025 PMC2711003

[57] Xin H Zhu J Miao H Gong Z Jiang X Feng X. Adenovirus-mediated CCR7 and BTLA overexpression enhances immune tolerance and migration in immature dendritic cells. BioMed Res Int. (2017) 2017:3519745. 10.1155/2017/3519745 28393074 PMC5368407

[58] Iwata A Watanabe N Oya Y Owada T Ikeda K Suto A. Protective roles of B and T lymphocyte attenuator in NKT cell-mediated experimental hepatitis. J Immunol. (2010) 184:127–33. 10.4049/jimmunol.0900389 19949073

[59] Miller ML Sun Y Fu YX. Cutting edge: B and T lymphocyte attenuator signaling on NKT cells inhibits cytokine release and tissue injury in early immune responses. J Immunol. (2009) 183:32–6. 10.4049/jimmunol.0900690 19535622

[60] Sekar D Govene L Del Rio ML Sirait-Fischer E Fink AF Brune B. Downregulation of BTLA on NKT cells promotes tumor immune control in a mouse model of mammary carcinoma. Int J Mol Sci. (2018) 19:E752. 10.3390/ijms19030752 29518903 PMC5877613

[61] Fourcade J Sun Z Pagliano O Guillaume P Luescher IF Sander C. CD8(+) T cells specific for tumor antigens can be rendered dysfunctional by the tumor microenvironment through upregulation of the inhibitory receptors BTLA and PD-1. Cancer Res. (2012) 72:887–96. 10.1158/0008-5472.CAN-11-2637 22205715 PMC3288235

[62] Serriari NE Gondois-Rey F Guillaume Y Remmerswaal EB Pastor S Messal N. B and T lymphocyte attenuator is highly expressed on CMV-specific T cells during infection and regulates their function. J Immunol. (2010) 185:3140–8. 10.4049/jimmunol.0902487 20693422

[63] Tian C Liu YG Yan JK Liu SD Zhao ST Wang HW. B- and T-lymphocyte attenuator/herpes virus entry mediator as early indicators for acute rejection following kidney transplantation. Transplant Proc. (2013) 45:157–62. 10.1016/j.transproceed.2012.10.019 23375291

[64] Cai G Nie X Li L Hu L Wu B Lin J. B and T lymphocyte attenuator is highly expressed on intrahepatic T cells during chronic HBV infection and regulates their function. J Gastroenterol. (2013) 48:1362–72. 10.1007/s00535-013-0762-9 23532637

[65] Nan XP Zhang Y Yu HT Li Y Sun RL Wang JP. Circulating CD4+CD25high regulatory T cells and expression of PD-1 and BTLA on CD4+ T cells in patients with chronic hepatitis B virus infection. Viral Immunol. (2010) 23:63–70. 10.1089/vim.2009.0061 20121403

[66] Hou JL Lai W. The guideline of prevention and treatment for chronic hepatitis B: a 2015 update. Zhonghua Gan Zang Bing Za Zhi. (2015) 23:888–905. 10.3760/cma.j.issn.1007-3418.2015.12.002 26739464 PMC12677373

[67] Chen J Wang L Fu Y Li Y Bai Y Luo L. The co-inhibitory pathway and cellular immune imbalance in the progress of HBV infection. Hepatol Int. (2014) 8:55–63. 10.1007/s12072-013-9464-x 26202406

[68] Yu HJ Li J Shen Q Expression of BTLA on peripheral lymphocytes in patients with primary carcinoma of the liver. Zhejiang Med. (2012) 34:1563–5.

[69] Liver Failure and Artificial Liver Group CSoID, Chinese Medical Association; Severe Liver Disease and Artificial Liver Group, Chinese Society of Hepatology, Chinese Medical Association Guideline for diagnosis and treatment of liver failure (2018). J Clin Hepatol. (2019)35:38–44. 10.3969/j.issn.1001-5256.2019.01.007

[70] Arroyo V Moreau R Jalan R Gines P Study E-CCC. acute-on-chronic liver failure: a new syndrome that will re-classify cirrhosis. J Hepatol. (2015) 62(1 Suppl):S131–43. 10.1016/j.jhep.2014.11.045 25920082

[71] Ohnishi H Sugihara J Moriwaki H Muto Y. [Acute-on-chronic liver failure]. Ryoikibetsu Shokogun Shirizu. (1995):217–9. 8749457

[72] Sarin SK Kumar A Almeida JA Chawla YK Fan ST Garg H. Acute-on-chronic liver failure: consensus recommendations of the Asian Pacific association for the study of the liver (APASL). Hepatol Int. (2009) 3:269–82. 10.1007/s12072-008-9106-x 19669378 PMC2712314

[73] Anand AC Dhiman RK. Acute on chronic liver failure-what is in a 'definition'? J Clin Exp Hepatol. (2016) 6:233–40. 10.1016/j.jceh.2016.08.011 27746620 PMC5052400

[74] Han L Wang W Fang Y Feng Z Liao S Li W. Soluble B and T lymphocyte attenuator possesses antitumor effects and facilitates heat shock protein 70 vaccine-triggered antitumor immunity against a murine TC-1 cervical cancer model *in vivo*. J Immunol. (2009) 183:7842–50. 10.4049/jimmunol.0804379 19923459

[75] Cao D Xu H Guo G Ruan Z Fei L Xie Z. Intrahepatic expression of programmed death-1 and its ligands in patients with HBV-related acute-on-chronic liver failure. Inflammation. (2013) 36:110–20. 10.1007/s10753-012-9525-7 22895698

